# The study on cuproptosis in Alzheimer’s disease based on the cuproptosis key gene *FDX1*

**DOI:** 10.3389/fnagi.2024.1480332

**Published:** 2024-12-16

**Authors:** Guilin Chen, Erwei Xi, Xiaozhen Gu, Huili Wang, Qiqiang Tang

**Affiliations:** ^1^Department of Neurology, Yijishan Hospital, The First Affiliated Hospital of Wannan Medical College, Wuhu, Anhui, China; ^2^Department of Neurology, Provincial Hospital Affiliated to Anhui Medical University, Hefei, Anhui, China; ^3^Institute of Food and Biological Engineering, Hefei University of Technology, Hefei, Anhui, China; ^4^Department of Neurology, The First Affiliated Hospital of University of Science and Technology of China, Hefei, Anhui, China

**Keywords:** Alzheimer’s disease, cuproptosis, *FDX1*, *DLAT*, *DLST*

## Abstract

**Background:**

Alzheimer’s disease (AD) is a neurodegenerative disorder characterized by memory and cognitive impairments. Previous studies have shown neuronal death in the brains of AD patients, but the role of cuproptosis and its associated genes in AD neurons remains unclear.

**Methods:**

Intersection analysis was conducted using the AD transcriptome dataset GSE63060, neuron dataset GSE147528, and reported cuproptosis-related genes to identify the cuproptosis key gene *FDX1* highly expressed in AD. Subsequently, cell experiments were performed by treating SH-SY5Y cells with Aβ_25-35_ to establish AD cell model. The real-time reverse transcriptase-polymerase chain reaction (RT-qPCR) and western blotting (WB) assays were employed to detect the expression levels of *FDX1*, *DLAT*, and *DLST*. Cell proliferation was analyzed by counting Kit-8 (CCK8), mitochondrial ROS levels were analyzed using flow cytometry. shRNA was used to downregulate *FDX1* expression, followed by repetition of the aforementioned experiments. Clinical experiments utilized qPCR to detect *FDX1* mRNA levels in peripheral venous blood of patients, and analyzed *FDX1* expression differences in different *APOE* genotypes of AD patients. Finally, a protein–protein interaction (PPI) network of *FDX1* was constructed based on the GeneMANIA database, immune infiltration analysis was conducted using R language, and transcription factors prediction for *FDX1* was performed based on the ENCODE database.

**Results:**

The cuproptosis key gene *FDX1* showed significantly higher expression in peripheral blood and neuron models of AD compared to non-AD individuals, with significantly higher expression in *APOE ε4/ε4* genotype than other *APOE* genotype of AD patients. Knockdown of *FDX1* expression reduced the lipidation levels of *DLAT* and *DLST* in neurons, alleviated ROS accumulation in mitochondria, improved cell viability, and mitigated cuproptosis. Immune infiltration analysis results indicated a high enrichment of peripheral blood γδ-T lymphocytes in AD, and *FDX1* was significantly associated with the infiltration of four immune cells and may be regulated by three transcription factors.

**Conclusion:**

The cuproptosis key gene *FDX1* is highly expressed in AD and may promote cuproptosis in AD neurons by regulating the lipidation levels of *DLAT* and *DLST*, thereby participating in the onset and development of AD. This provides a potential target for the diagnosis and treatment of AD.

## Introduction

Alzheimer’s disease is currently the most common type of dementia, accounting for approximately 60–70% of all dementia cases (Gao et al., 2022; Huang, 2023), affecting around 47 million people worldwide (Oboudiyat et al., 2013). Its clinical features include impairments in executive and visuospatial functions, as well as short-term memory deficits (Scheltens et al., 2021). The exact etiology of AD remains incompletely understood. Currently, the leading pathological hypotheses revolve around the deposition of amyloid-*β* (Aβ) forming senile plaques and abnormal p-Tau protein forming neurofibrillary tangles, leading to neuronal loss and death (Bharadwaj et al., 2009; Van der Kant et al., 2020). However, numerous targeted drug clinical trials globally based on these hypotheses have ended in failure (Lam et al., 2013; Byun et al., 2015), and there is still no effective treatment available to slow down, treat, or reverse AD (Lam et al., 2013; Byun et al., 2015). The exact causes and pathogenic mechanisms of AD remain to be further elucidated, with research focusing on alternative or innovative fields (Lai et al., 2022).

Long-term observational studies have found widespread neuronal loss in the brains of AD patients. Neuronal death is the direct cause of neurodegenerative changes, resulting in irreversible damage to the nervous system, leading to memory deficits and cognitive impairments (Yan et al., 2021; Duan et al., 2016). However, previous relevant studies mainly focused on apoptosis (Mangalmurti and Lukens, 2022).

Regulated cell death (RCD), as determined by genes, plays a crucial role in the orderly active death of cells, regulating homeostasis (Peng et al., 2022). As research by domestic and international scholars deepens, regulated cell death modes such as ferroptosis, pyroptosis, and cuproptosis have been shown to play important roles in diseases (Wang et al., 2023).

In March 2022, Tsvetkov et al. first proposed and confirmed cuproptosis as a novel form of cell death (Tsvetkov et al., 2022). Unlike other known forms of cell death such as apoptosis, pyroptosis, and necroptosis, cuproptosis, similar to ferroptosis and zinc-induced cell death, is a regulated cell death induced by metal ion overload. When copper directly binds to the lipoyl moiety of the tricarboxylic acid cycle, it triggers the loss of iron–sulfur cluster proteins, protein toxicity stress, and ultimately leads to cell death (Tsvetkov et al., 2022). Among them, *FDX1* serves a key regulator of cuproptosis and a direct target of elesclomol (ES), a copper ionophore that induces cuproptosis by promoting oxidative stress (Tsvetkov et al., 2019). *FDX1* is directly related to protein lipoylation and significantly associated with ATP, ROS, etc. (Zhang et al., 2021; Zhang et al., 2022), thereby playing a crucial role in cuproptosis. Knocking out the key upstream regulator *FDX1* of lipoylation proteins or lipoylation-related enzymes can block cuproptosis (Tsvetkov et al., 2022).

There have been preliminary studies exploring the correlation between cuproptosis-related genes and immune cells in AD (Nie et al., 2023; Zhang et al., 2023), and experimental evidence showing that Cu overload can induce extensive neuronal cell death in the hippocampus of mice (Zhang et al., 2023), but the role of cuproptosis in the pathogenesis of AD is still unclear.

Based on this, we reasonably speculate that the cuproptosis mechanism may be involved in the pathological process of AD. However, the role of cuproptosis and its related gene regulation in AD remains to be determined. This study aims to screen for differentially expressed genes related to cuproptosis in AD neurons by biological information technology, verify their expression in peripheral blood and neurons of AD, and explore their impact on the activity of AD neurons to provide insights for effective treatment strategies.

## Materials and methods

### Data download and processing

The transcriptome dataset GSE63060 for AD patients (*n* = 145) and healthy subjects (*n* = 184) was downloaded from the GEO database^1^ and analyzed for differential expression genes (DEGs) using the Limma (Ritchie et al., 2015) package in R language. The single-cell dataset GSE147528 was also obtained and processed using the Seurat package version 3.2.2. Principal component analysis (PCA) was performed, and key principal components (PCs) were selected by executing JackStraw and PCEIbowPlot functions. The FindAllMarkers function in Seurat was used to accurately identify genes specific to each cell subtype, followed by UMAP algorithm-based cell clustering and visualization analysis using the RunUMAP method. Annotation of marker genes was done using the singleR package, and feature calibration was performed using CellMarker. The corrected DEGs, neuron cell-related genes, and collected cuproptosis genes were intersected using an online Venn diagram tool to obtain the differential expression cuproptosis-related genes (DE-CRG): *FDX1*.

### Cell culture and transfection

The human neuroblastoma cell line SH-SY5Y was obtained from the Cell Bank of the Chinese Academy of Sciences. Cells were cultured in DMEM medium supplemented with 10% fetal bovine serum and 1% antibiotics (penicillin–streptomycin mixture) at 37°C and 5% CO_2_.

Given that the generation, aggregation, and deposition of Aβ are considered pivotal initiating events in the pathological cascade of AD, with the Aβ_25-35_ peptide segment being one of the most neurotoxic fragments (Wang et al., 2023), it is capable of inducing oxidative cellular damage, increasing the production of reactive oxygen species (ROS) within cells, and leading to apoptosis, thereby simulating key pathological processes of AD in cellular models (Arispe et al., 1993; Changhong et al., 2021; Limón et al., 2009). Consequently, this study employed a concentration of 20 μmol/L of Aβ_25-35_ to treat SH-SY5Y cells for 36 h in order to establish an AD cellular model.

Short hairpin RNA (shRNA) targeting *FDX1* (pGPU6-shFDX1-1 and pGPU6-shFDX1-2) were purchased from Shanghai Jierui Biotechnology Co., Ltd., containing two different *FDX1* shRNA sequences (shFDX1-1: 5’-GGACAAUAUGACUGUUCGAGU-3’, shFDX1-2: 5’-AGUUGGUGAUUCUCUGCUAGA-3’). The plasmids were transfected into SH-SY5Y cells (density: 2 × 10^5/ml in 6-well plates) using Lipofectamine 2000 Reagent (Life Technologies, Carlsbad, CA, USA) according to the manufacturer’s instructions, with pGPU6 empty vector used as a negative control.

### Clinical data and sample collection

#### Study subjects

The study subjects were patients admitted to the Neurology Department of Anhui Provincial Hospital from 2021 to 2023. The inclusion criteria for the disease group followed the core clinical criteria for AD dementia from the National Institute on Aging and Alzheimer’s Association (NIAAA-2011) (McKhann et al., 2011), and 30 typical AD patients were included based on cerebrospinal fluid and imaging biomarkers. The control group consisted of 20 hospitalized patients with normal cognitive function during the same period. All subjects signed informed consent forms for the study and received approval from the hospital’s Medical Ethics Committee, with ethics approval number: 2019KY Ethics Review No. 79.

#### Data collection

Patient information including name, gender, age, medical history, laboratory tests, and MMSE scores were collected through electronic medical records and on-site questionnaires. MMSE scores of ≤19 (illiterate), ≤22 (primary education), and ≤ 27 (middle school education and above) were considered indicative of cognitive impairment.

#### Sample collection

Peripheral venous blood was collected from all subjects around 6 a.m. on the day after admission (fasting for at least 8 h) into EDTA-K2 anticoagulant tubes. Within 2 h, the blood was aliquoted into centrifuge tubes (EP tubes) at 400 μL per tube, stored in −80°C, and avoided repeated freeze–thaw cycles.

### Real-time reverse transcriptase-polymerase chain reaction

Total RNA was extracted and purified from cells/whole blood using TRIzol reagent according to the manufacturer’s instructions. The extracted RNA was reverse transcribed into cDNA, followed by RT-qPCR amplification normalizing all samples to GAPDH. The gene primers were synthesized by Shanghai Jierui Biotechnology Co., Ltd., which sequences are listed in Table 1. The corresponding Ct values and melting curves were recorded after the reaction, and the 2^-ΔΔCt^ method was used for relative quantitative analysis of the target genes (Yildiz Gulhan et al., 2022).

**Table 1:** Sequence of gene primers.

Primer names	Primer sequences (5^’^-3^’^) Product length	
*GAPDH*	Forward:GAGAAGGCTGGGGCTCATTT	231 bp
	Reverse:AGTGATGGCATGGACTGTGG	
*FDX1*	Forward:ACCACGCTGGGTCCCG	250 bp
	Reverse:GTTCCCTCACATGCACCAAAGC	
*DLAT*	Forward:TGATGTCAGTGTTGCGGTCA	166 bp
	Reverse:CGTAAAAGTGCCACCCTGGA	
*DLST*	Forward:AGGTGGGAGAAAGCTGTTGG	230 bp
	Reverse:TCCCAAGAGGGAACACTGGA	

### Western blotting

Cells were separated using a cell scraper and then incubated on ice for 30 min. Protein lysates were prepared by adding 4:1 volume ratio of 5× reducing protein loading buffer, followed by denaturation in a boiling water bath for 15 min. Total protein content was separated by SDS-PAGE electrophoresis, and 20 μg of protein samples were loaded. Electrophoresis was conducted initially at 80 V for 20 min and then increased to 120 V for an additional 60 min. Subsequently, proteins were transferred onto PVDF membranes at 25 V for 30 min. After blocking in skim milk, the membranes were incubated overnight at 4°C with primary antibodies against the target proteins (*FDX1*, *DLAT*, and *DLST*) and the internal reference (*GAPDH*). The next day, the membranes were incubated with goat anti-rabbit HRP-conjugated secondary antibodies and goat anti-mouse HRP-conjugated secondary antibodies for 30 min. After incubation, the membranes were washed and visualized, and band grayscale values were calculated using Image J imaging analysis software.

### Cell proliferation assay

The effect of *FDX1* downregulation on cell viability was determined by CCK-8. SH-SY5Y cells and transfected cells were treated at 0, 24, 48, and 72 h. CCK-8 and serum-free essential culture medium were mixed at a 1:10 volume ratio, and 100 μL of the mixture was added to each well. Cells were then incubated at 37°C and 5% CO_2_ for 2 h, and absorbance at 450 nm wavelength was measured using a microplate reader.

### Flow cytometry

Total mitochondrial ROS in SH-SY5Y cells was detected using the DCFH-DA probe (Beyotime Biotechnology). Cells were incubated with diluted DCFH-DA probe (1 mL) in serum-free culture medium for 20 min, followed by three washes with serum-free culture medium. Fluorescence intensity was measured by flow cytometry at an excitation wavelength of 488 nm and an emission wavelength of 525 nm.

### Protein–protein interaction network

The PPI network of *FDX1* was automatically constructed on the GeneMANIA database^2^ online website, and functional and enrichment pathway analyses were also performed. The more connecting lines between proteins in the network diagram, the stronger the correlation.

### Immune infiltration analysis

Based on the gene expression profile of AD patients from the GSE63060 transcriptome dataset, the CIBERSORT algorithm was used to calculate the proportions of various cell types (*p <* 0.05). The “pheatmap” package was used to create a heatmap of 22 immune cell types, and the “corrplot” package was used to create a correlation heatmap to visualize the correlation between 22 different infiltrating immune cells. The relationship between infiltrating immune cells and *FDX1* was analyzed by Spearman rank correlation test in R package.

### Transcription factors prediction

*FDX1*-related transcription factors binding data were retrieved from the ENCODE database. Through bioinformatics analysis tools, the binding strength and differential expression patterns of transcription factors were compared to predict which transcription factors might affect *FDX1* expression.

### Statistical analysis

Statistical analysis was performed using SPSS 25.0 software. Count data were expressed as numbers of cases, and qualitative data were compared using the χ^2^ test. Quantitative data following a normal distribution were expressed as (x̄ ± s), and comparisons between two groups were performed using independent sample t-tests. Non-normally distributed data were expressed as median (upper quartile, lower quartile), and comparisons between two groups were performed using Mann–Whitney *U* test. *p*-value <0.05 was used as the filtering condition. Experimental data were plotted using GraphPad Prism 10.0.

## Results

### Dataset information and DE-CRG screening results

**Figure 1:**
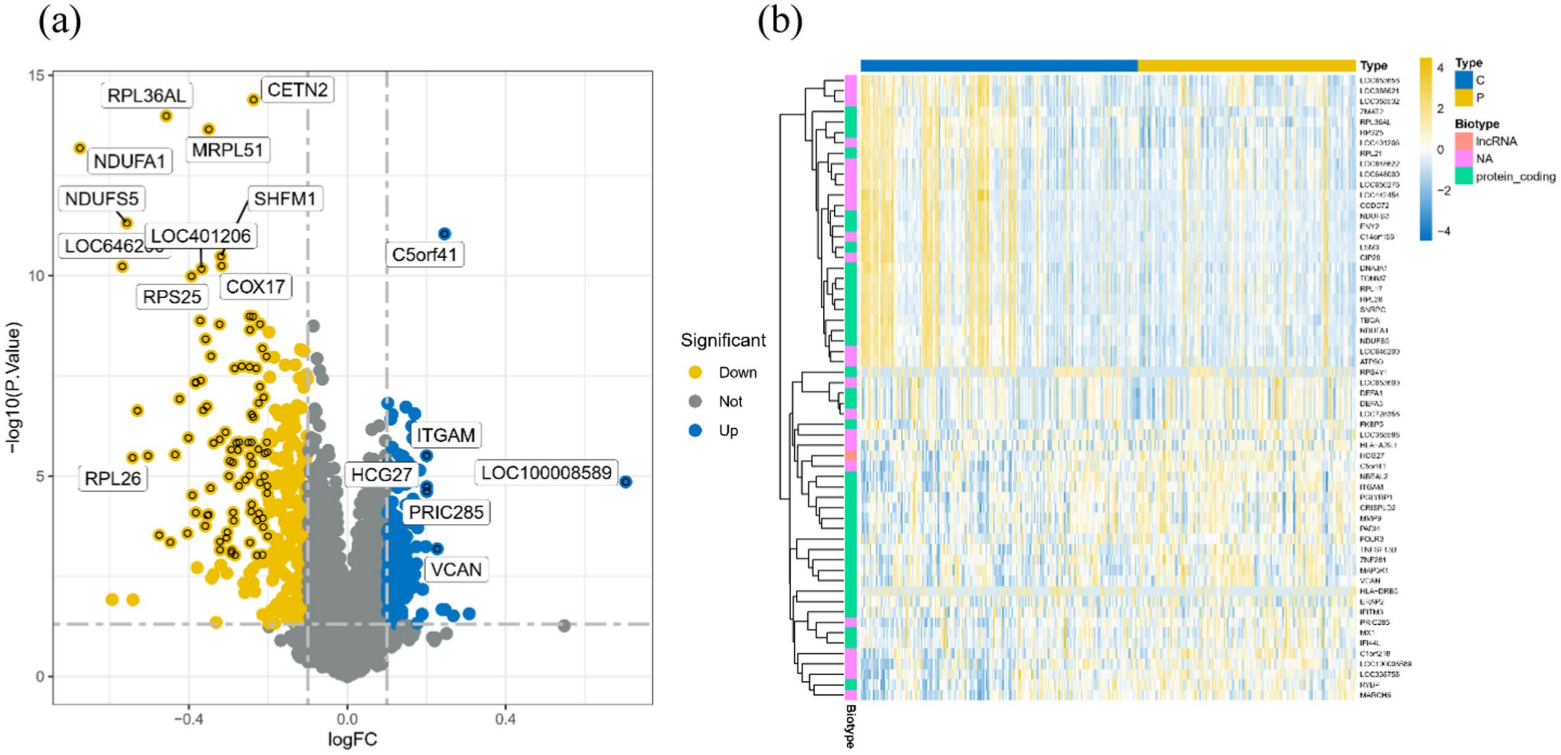
Volcano map and heat map of DEGs: **(A)** Volcano map of DEGs, blue for up-regulated gene, yellow for down-regulated gene, gray for no significant difference; **(B)** Heat maps of DEGs in different groups, blue for healthy controls and yellow for disease groups.

**Figure 2:**
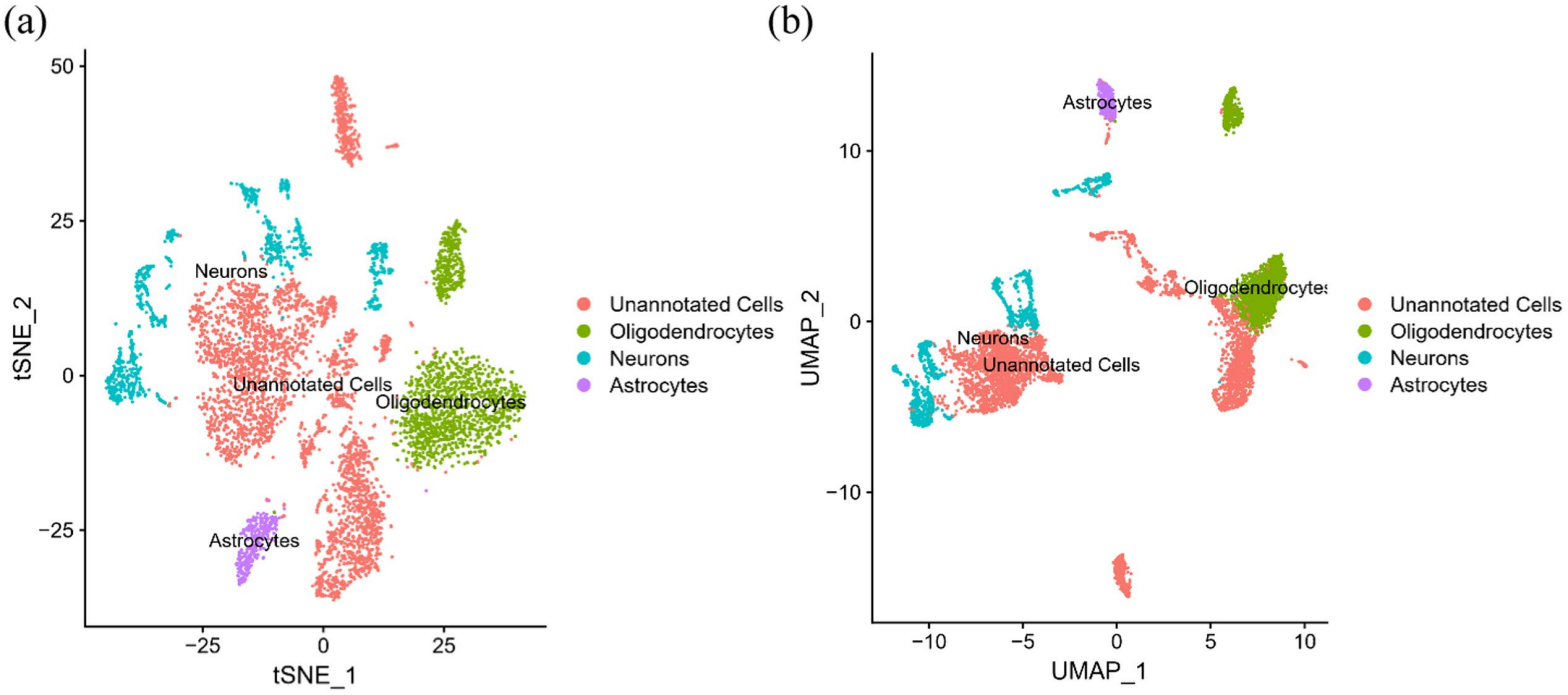
T-SNE and UMPA atlas: **(A)** T-SNE projection of 3,000 cells from all brain tissues; **(B)** UMPA projection of 3,000 cells from all brain tissues.

**Figure 3:**
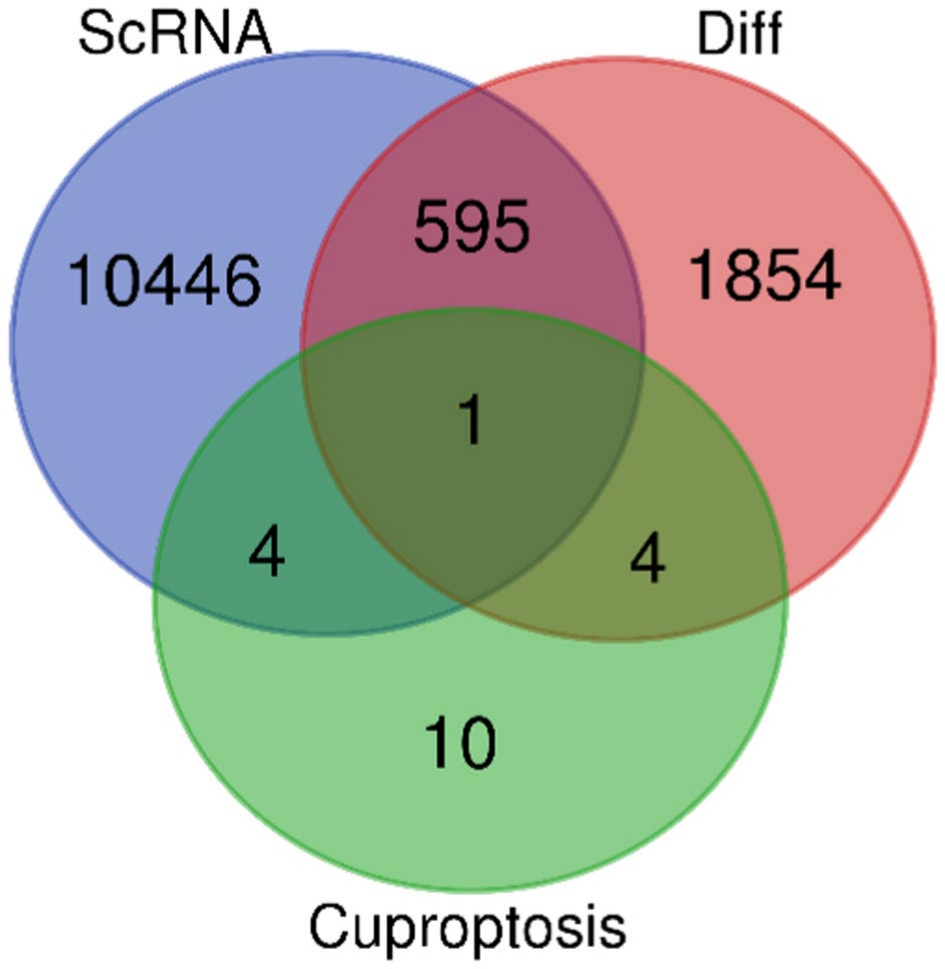
Venn diagram of the three data sets (DE-CRG is the cross part).

After careful screening, the original files of GSE63060 were downloaded. DEGs were filtered with criteria of *p <* 0.05 and |logFC| > 1, resulting in 21,684 genes meeting the requirements, with 11,043 genes upregulated and 10,641 genes downregulated. The volcano plot and heatmap of DEGs were generated using the ggplot2 and pheatmap packages (Figure 1). T-SNE and UMAP plots were used to visualize the expression distribution of neurons in clusters, demonstrating the heterogeneity of neurons in the GSE147528 dataset (Figure 2). Finally, a Venn diagram was constructed to intersect the processed DEGs, neuron cell-related genes, and cuproptosis-related genes, obtaining DE-CRG: *FDX1* (Figure 3).

### High expression of *FDX1* in AD model cells

**Figure 4:**
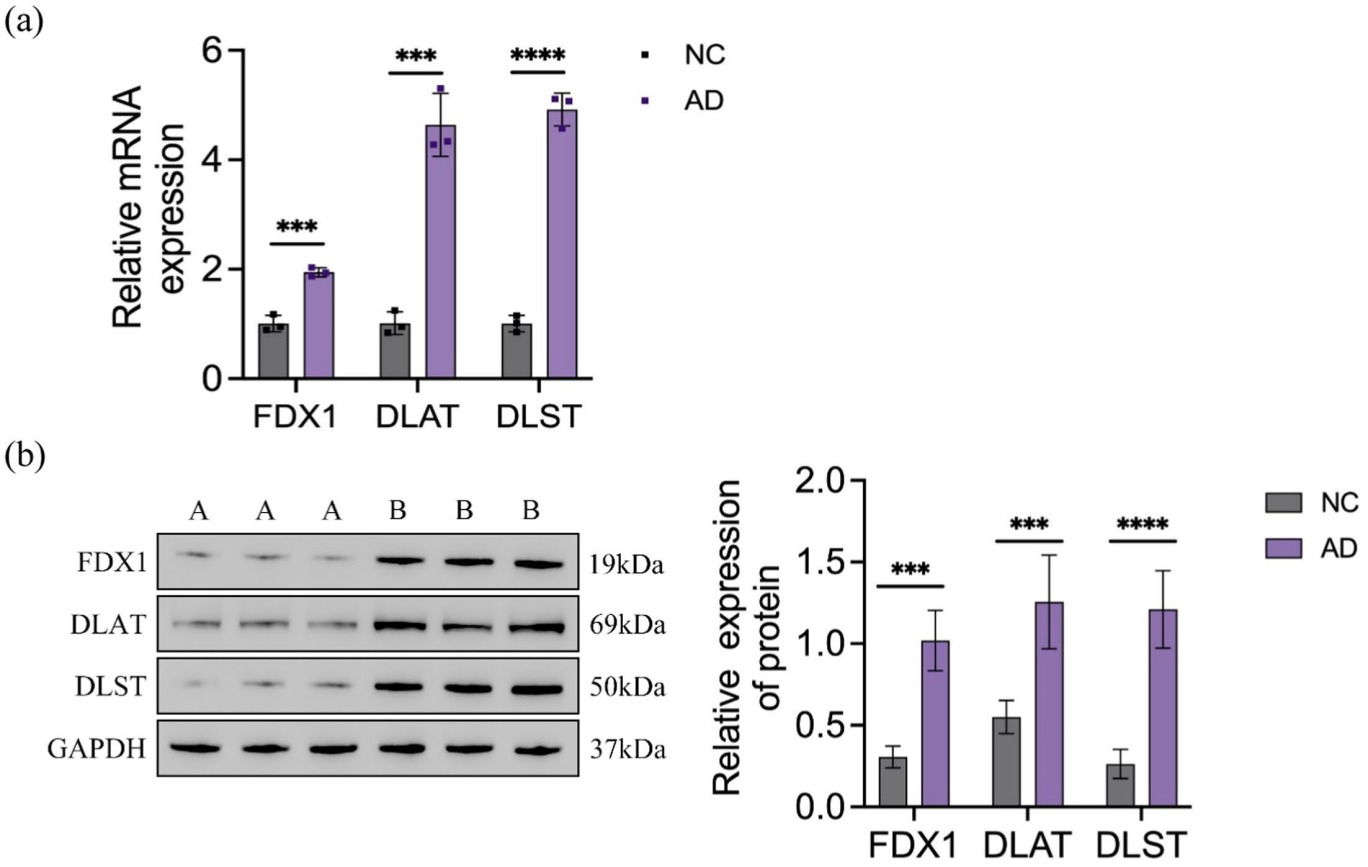
**(A)** Relative expression of *FDX1* mRNA, *DLAT* mRNA, *DLST* mRNA in NC and AD model SH-SY5Y cells; **(B)** Relative expression of *FDX1*, *DLAT*, *DLST* protein levels in NC and AD model SH-SY5Y cells (***p < 0.001, ****p < 0.0001). A: control group, B: model group.

The relative expression levels of genes and proteins in cells were detected using RT-qPCR and WB assays, with results depicted in Figure 4. In the Aβ_25-35_-induced AD model SH-SY5Y cells, the relative expression levels of *FDX1*, *DLAT*, and *DLST* mRNA and proteins were significantly increased compared to the control group.

### Decreased expression of *DLAT* and *DLST* after *FDX1* knockdown

**Figure 5:**
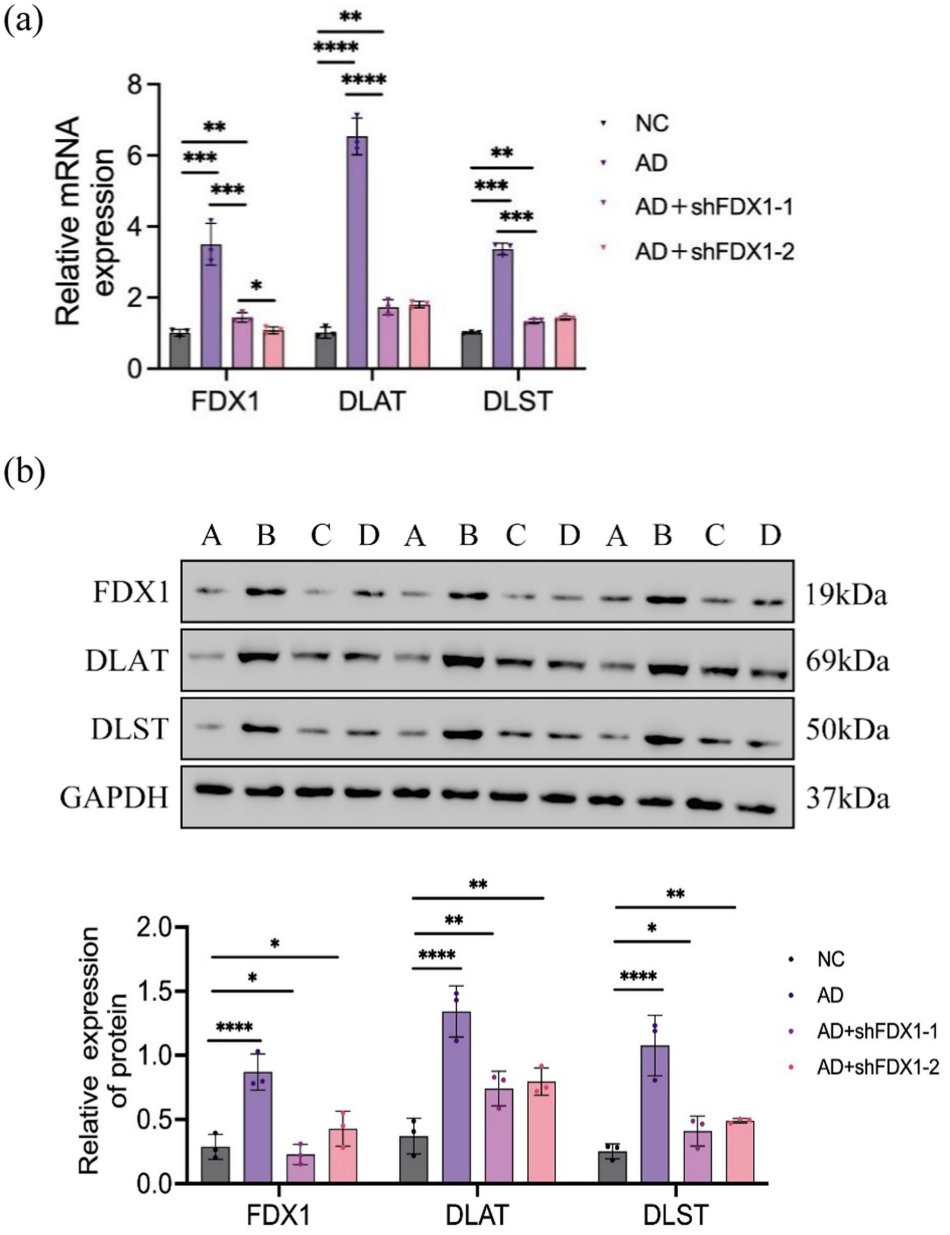
**(A)** Relative expression of *FDX1* mRNA, *DLAT* mRNA and *DLST* mRNA in SH-SY5Y cells of NC, AD, AD + shFDX1-1 and AD + shFDX1-2 models; **(B)** Relative expression of *FDX1*, *DLAT* and *DLST* in SH-SY5Y cells of NC, AD, AD + shFDX1-1 and AD + shFDX1-2 models. (*p < 0.05, **p < 0.01, ***p < 0.001, ****p < 0.0001). A: control group, B: model group, C: model group + shFDX1-1, D: model group + shFDX1-2.

After downregulation of *FDX1* expression, the relative expression levels of *DLAT* and *DLST* mRNA and proteins in the AD + shFDX1-1 and AD + shFDX1-2 model SH-SY5Y cells were markedly reduced compared to the control group, as shown in Figure 5.

### Increased cell proliferation activity after *FDX1* knockdown

**Figure 6:**
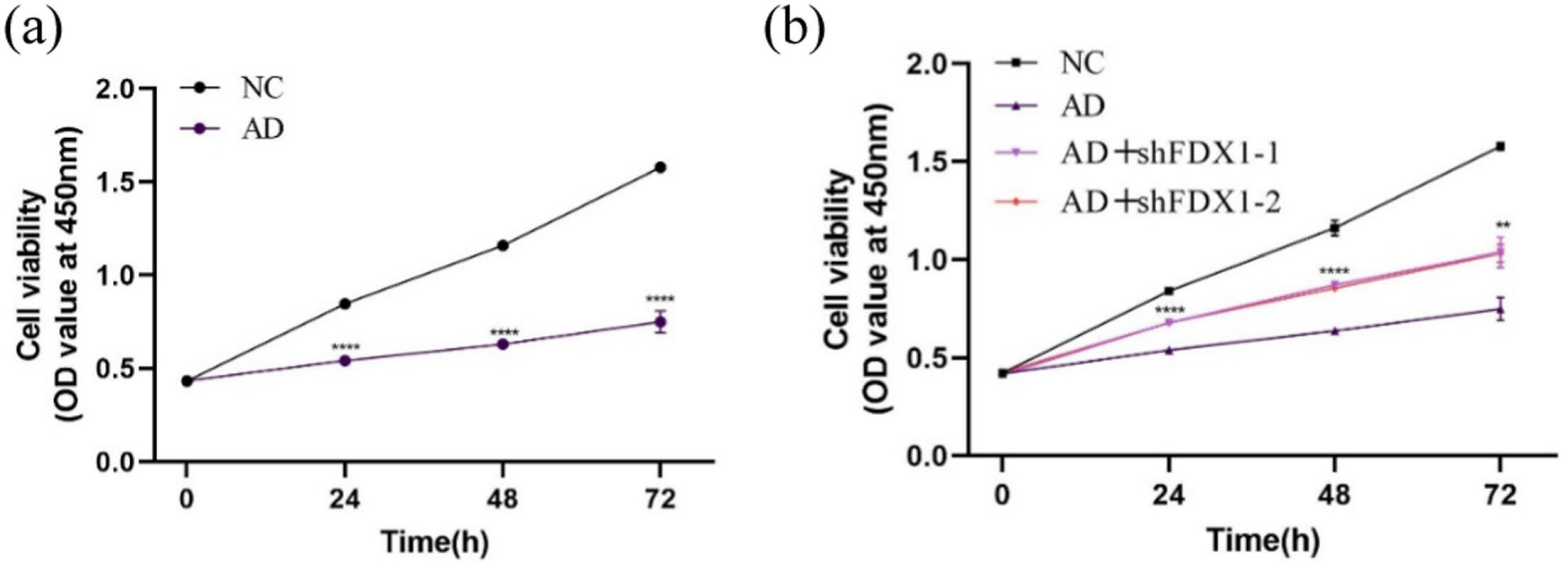
**(A)** The effect of Aβ_25-35_ on the viability of SH-SY5Y cells; **(B)** The effect of knocking down *FDX1* expression on the viability of SH-SY5Y cells in AD model. (**p < 0.01, ***p < 0.001, ****p < 0.0001).

Cell proliferation activity of SH-SY5Y cells was measured by CCK8 assay at 0, 24, 48, and 72 h. Compared to the control group, cell proliferation activity significantly decreased after Aβ_25-35_ treatment (Figure 6A). After *FDX1* knockdown, cell proliferation activity significantly increased compared to the non-knockdown group (Figure 6B). At 48 h, cell viability recovered to 71.41% (*p <* 0.0001).

### Reduced cell damage after *FDX1* mRNA knockdown

**Figure 7:**
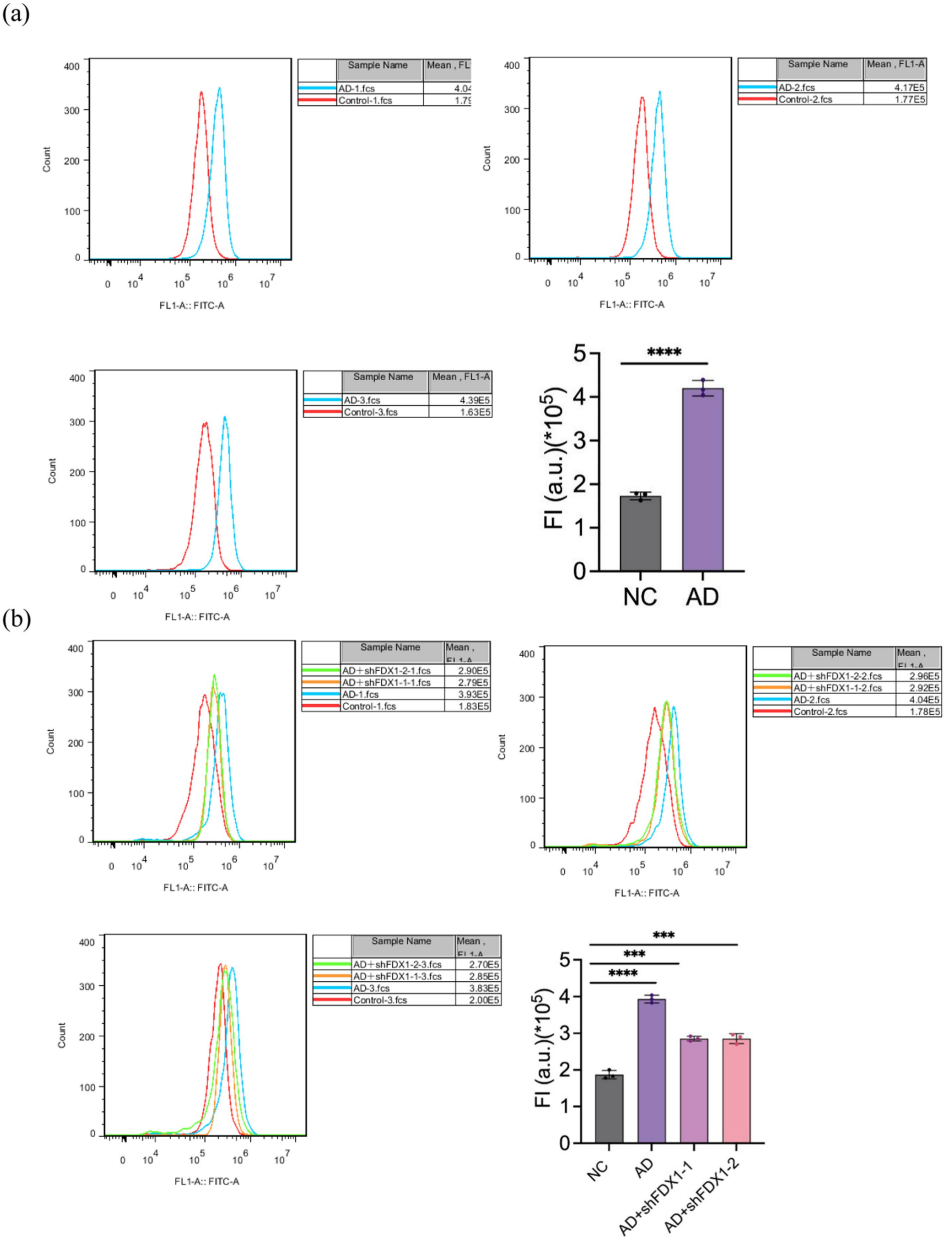
**(A)** Effect of Aβ_25-35_ on mitochondrial ROS content in SH-SY5Y cells. **(B)** The effect of knocking down *FDX1* expression on mitochondrial ROS content in SH-SY5Y cells of AD model. (***p < 0.001, ****p < 0.0001).

Mitochondrial ROS levels in SH-SY5Y cells were measured using flow cytometry. Compared to the control group, mitochondrial ROS accumulation in SH-SY5Y cells significantly increased after Aβ_25-35_ treatment (Figure 7A). After *FDX1* knockdown, mitochondrial ROS levels in SH-SY5Y cells decreased significantly (*p <* 0.001) (Figure 7B), indicating alleviated cell damage.

### Comparison of general information and *FDX1* expression in peripheral blood between AD and control group

**Figure 8:**
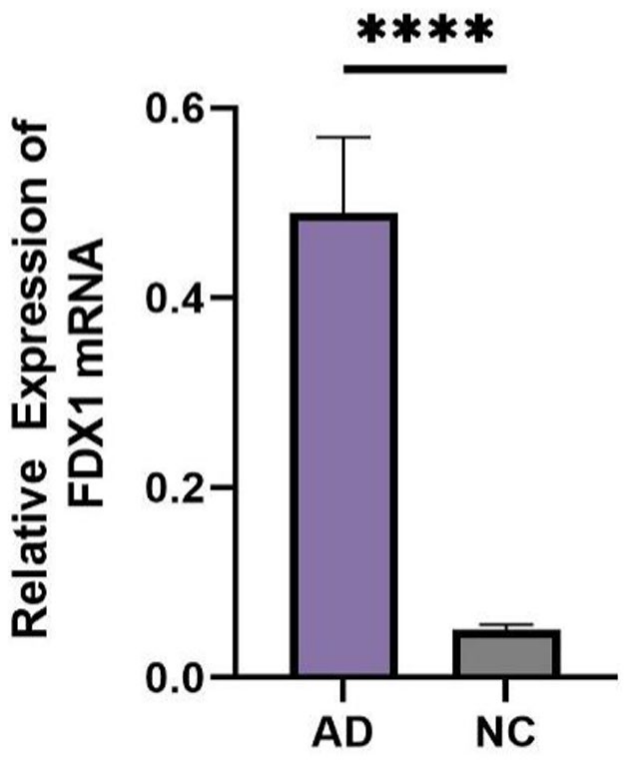
Difference in expression of *FDX1* mRNA between AD group and NC group (****p < 0.001).

There were statistical differences in gender, age, MMSE, and lipoprotein(a) (Lp(a)) between the two groups (*p <* 0.05) (Table 2). The relative expression of *FDX1* mRNA in the peripheral blood of AD patients was significantly increased compared to non-AD patients (*p* < 0.0001) (Figure 8).

**Table 2:** General data analysis of AD group and control group.

Profile		AD group (*n* = 30)	NC group (*n* = 20)	*t/x^2^/z*	*p*
Sex	Male	8	11	4.089	0.043
	Female	22	9		
Age		65.70 ± 8.66	56.40 ± 10.70	3.384	0.001
MMSE		17.00(9.00–20.75)	27.00(27.00–28.00)	−5.958	<0.001
Lp (a)(mg/L)		235.00(160.00–278.00)	95.50(63.25–155.50)	−2.032	0.042

### Comparison of *FDX1* expression among AD patients with different *APOE* genotypes

**Figure 9:**
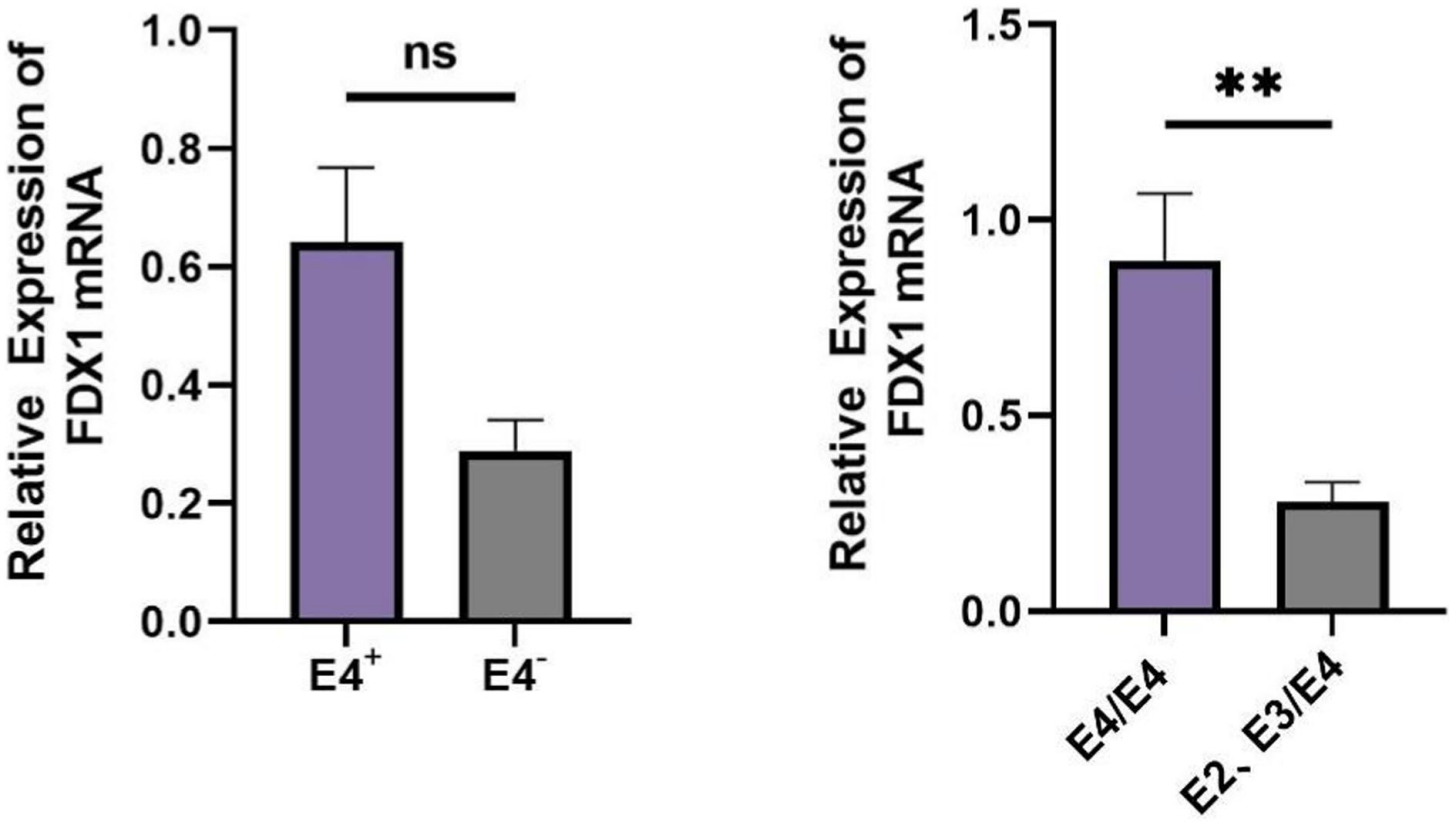
**(A)** Difference analysis of relative expression of *FDX1* mRNA in *APOE ε4^+^/APOE ε4^−^* of AD patients; **(B)** Difference analysis of relative expression of FDX1 mRNA in *APOE ε4/ε4* and *APOE ε2 ε3/ε4* of AD patients (**p < 0.01).

In AD patients, there was no statistical difference in *FDX1* mRNA relative expression levels between *APOE ε4^+^* and *APOE ε4^−^* individuals, but there was differential expression between *APOE ε4/ε4* and *APOE ε2*, *ε3/ε4* individuals (*p <* 0.05) (Figure 9). *FDX1* mRNA relative expression levels in peripheral blood of AD patients with the *APOE ε4/ε4* genotype were significantly higher.

### PPI analysis results of *FDX1*

**Figure 10:**
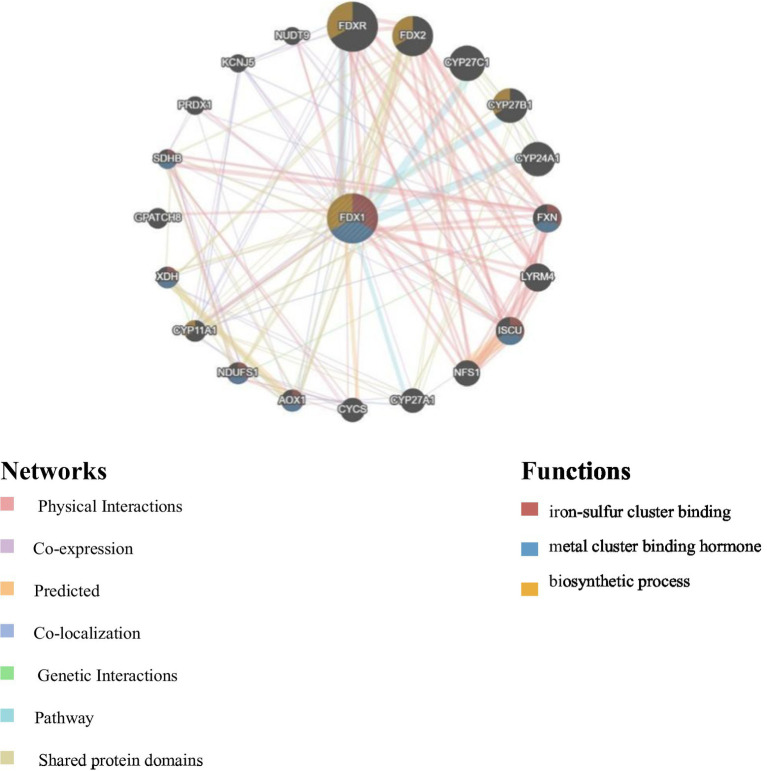
PPI network of *FDX1.* The colors in the circle represent the different functions of the proteins corresponding to each gene, and the colors of the lines represent the different interactions between the proteins. The more lines there are, the closer the connections are.

The PPI network showed that *FDX1* primarily interacts with *FDXR*, *FDX2*, *CYP27C1*, *CYP27B1*, and *CYP24A1*. Functional predictions indicated that *FDX1* is involved in hormone biosynthesis along with *FDXR*, *FDX2*, and *CYP27B1*, and also participates in the binding process of iron–sulfur clusters and metal clusters (Figure 10).

### High infiltration of γδ-T cells in AD

**Figure 11:**
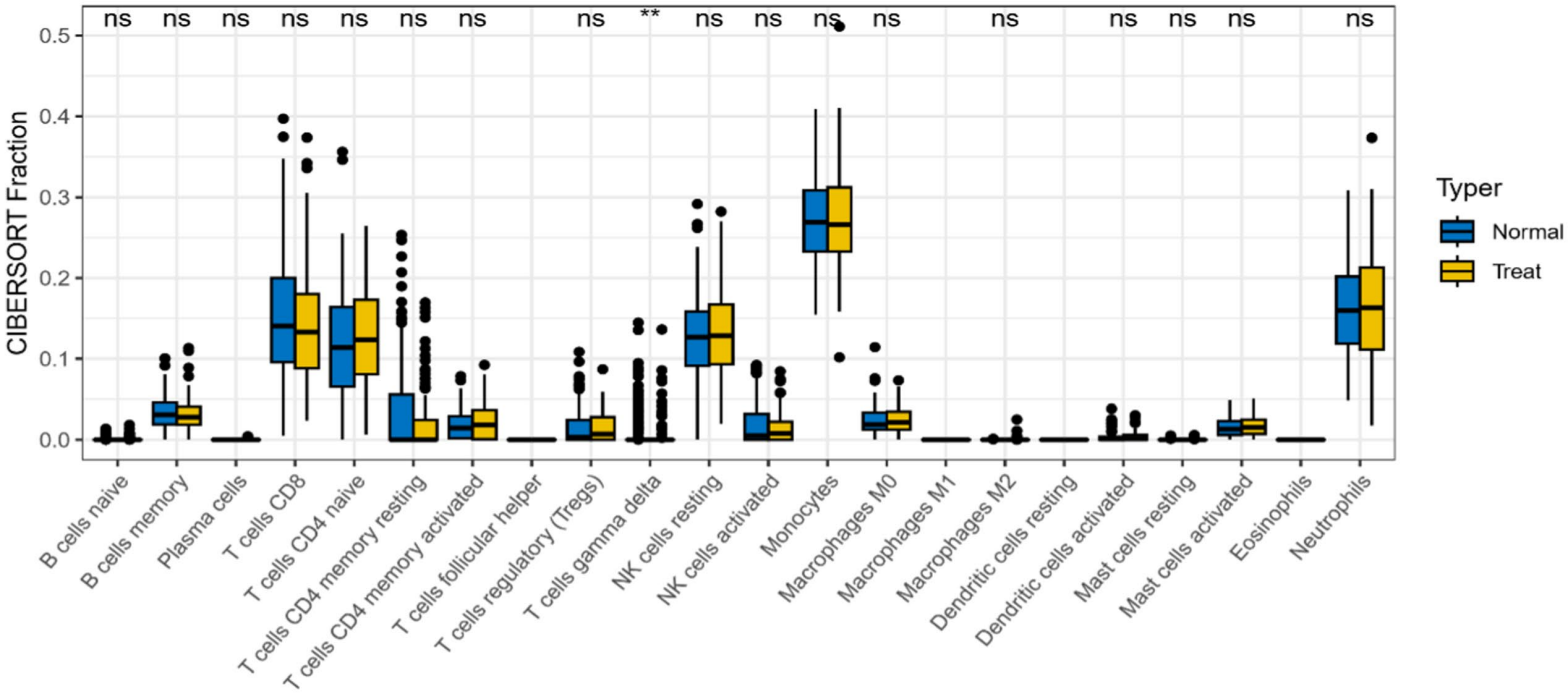
Differences in expression levels of immune cells in AD group and control group (*p < 0.05, **p < 0.01).

In differential analysis of immune cell infiltration (Figure 11), there was a significant difference in γδ-T cell infiltration expression between AD patients and healthy controls (*p <* 0.01). γδ-T cells were enriched and increased in AD patients, indicating possible immune inflammatory reactions in AD.

### Significant correlation between *FDX1* and four immune cells

**Figure 12:**
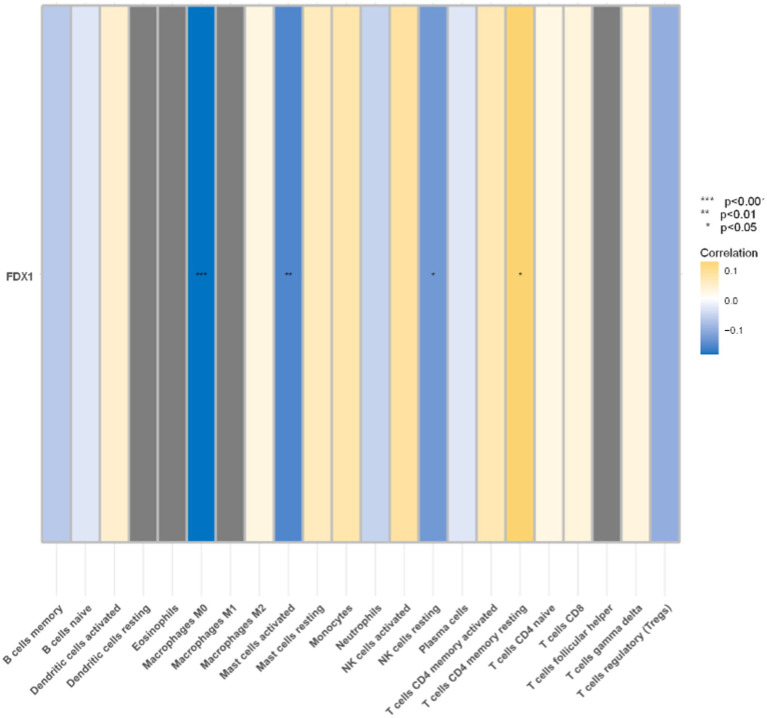
Correlation analysis between immune cells and *FDX1* (*p < 0.05,**p < 0.01,***p < 0.001). Blue represents negative correlation, yellow represents positive correlation.

*FDX1* were significantly correlated with four immune cells in the analysis of the correlation between 22 immune cells and it (Figure 12). Specifically, *FDX1* was negatively correlated with M0 macrophages (*p <* 0.001), activated mast cells (*p <* 0.01), and resting NK cells (*p <* 0.05), and positively correlated with CD4 memory resting T cells (*p <* 0.05).

### Existence of three transcription factors for *FDX1*

**Figure 13:**
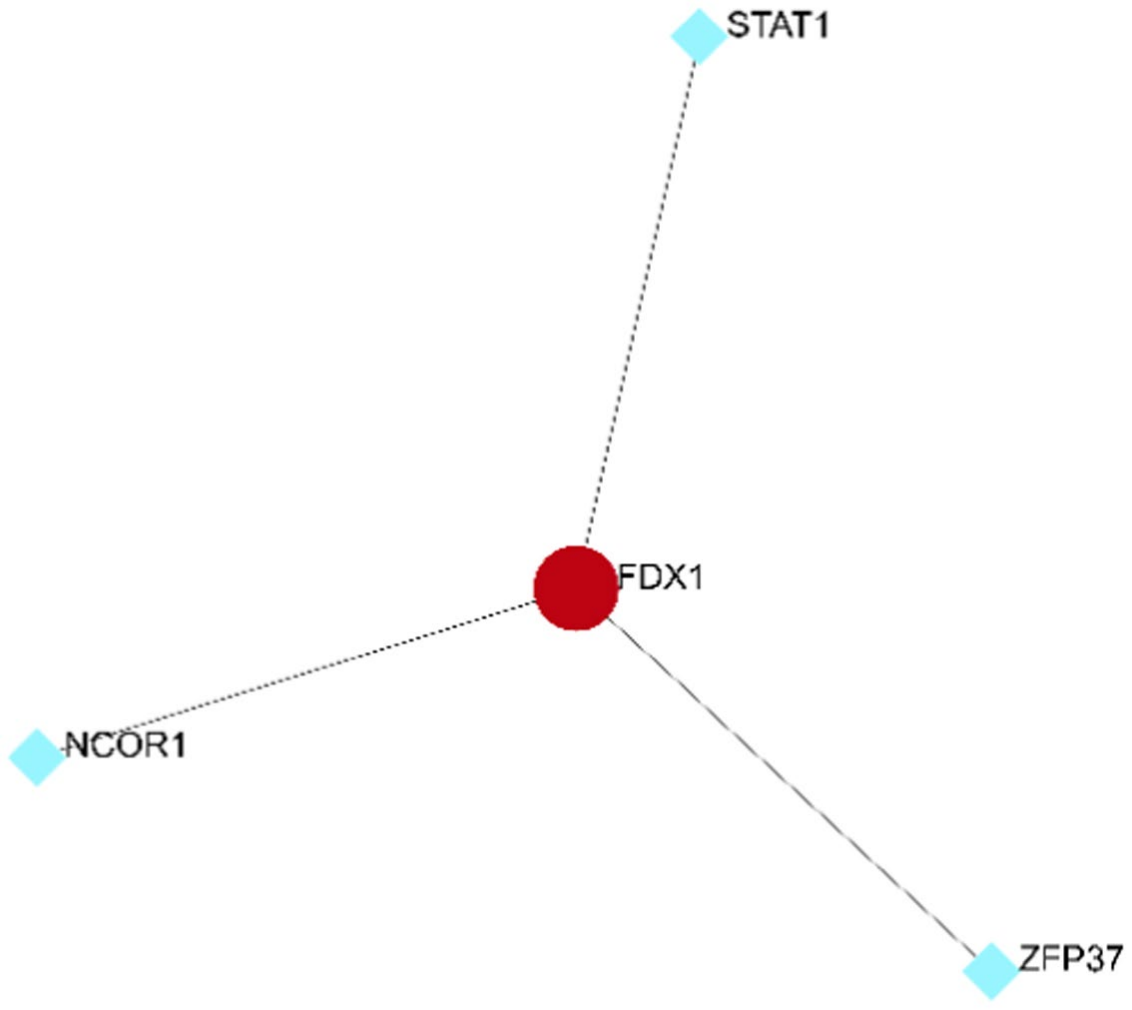
Transcription factors prediction of *FDX1.*

Based on the ENCODE database, three transcription factors related to *FDX1* were predicted (Figure 13): Signal Transducer and Activator of Transcription 1 (*STAT1*), Zinc Finger Protein 37 (*ZFP37*), and Nuclear Receptor Corepressor 1 (*NCOR1*).

## Discussion

Currently, research on cuproptosis in AD is still in its infancy, lacking direct experimental validation of the relevance between cuproptosis and AD. Therefore, this study conducted preliminary research on the cuproptosis mechanism in AD based on *FDX1* by biological information technology and experimental verification. The results indicate that *FDX1* is highly expressed in AD neurons. After downregulation of *FDX1* expression, the expression of acylated proteins *DLAT* and *DLST* in cuproptosis also decreased. Neuronal oxidative stress damage was significantly alleviated, and proliferation activity was markedly improved. Subsequently, using peripheral blood samples from some AD clinical patients, we found that *FDX1* was also highly expressed in AD patients, with higher expression levels in patients carrying the *APOE ε4/ε4* genotype. The experiments verified the high expression of *FDX1* in the peripheral and central nervous systems of AD patients, which is associated with the *APOE ε4/ε4* risk gene and may regulate neurons through the cuproptosis pathway. Inhibition of *FDX1* expression can alleviate cuproptosis in AD neurons, thereby delaying the progression of AD.

This study’s results demonstrate that after downregulation of *FDX1* expression, the expression of *DLAT* and *DLST* also decreases, potentially affecting the cuproptosis process through the *FDX1*-*DLAT/DLST* regulatory axis (Belloy et al., 2019), consistent with previous research findings. The loss of *FDX1* disrupts the acylation of proteins such as *DLAT* and *DLST* (Nie et al., 2023), resulting in loss of their ability to bind to copper, thus easing the cuproptosis process in neurons. Therefore, subsequent gene expression regulation can be achieved through *FDX1* transcription factors *STAT1*, *ZFP37*, and *NCOR1*, providing new strategies for AD treatment.

*APOE* mainly functions as a lipid transport protein, playing a key role in certain transport between neurons (Yamazaki et al., 2019). It can also affect the development of AD by blocking Aβ clearance, accelerating Aβ aggregation, influencing Tau pathology and Tau-mediated neurodegeneration, and damaging synaptic integrity and plasticity (Komai et al., 2024; Strittmatter et al., 1993). In 1993, *APOE* isoforms were first confirmed as risk factors for AD (Strittmatter and Roses, 1996), among which the *ε4* isoform increases the risk of developing AD and lowers the age of onset. One *ε4* allele can increase the risk of developing AD by 2- to 4-fold, while homozygosity for the allele can increase the risk to 8–12 times (Gao et al., 2022; Strittmatter and Roses, 1996).

This study showed that *FDX1* is more highly expressed in patients with AD carrying the *APOE ε4/ε4* genotype, suggesting that *FDX1* may interact with the *APOE ε4/ε4* genotype, jointly affecting the progression of the disease.

Clinical data show differences in age, gender, and lipoprotein between the two groups of patients. Age is one of the most significant risk factors for cognitive decline in AD (Liu et al., 2024). The older the age, the higher the probability of developing AD. Women are more likely to develop AD than men (Alzheimer’s Disease International, 2018)^,^ with a 19–29% higher incidence rate (ADI, n.d.), possibly due to women having a higher Tau protein load (Buckley et al., 2019; Babapour Mofrad et al., 2020), measured at higher levels of AD pathology (Oveisgharan et al., 2018; Biechele et al., 2024). Related experiments have shown that the effects of Cu on memory function and oxidative stress in rats are also gender-related, with slightly greater effects observed in female rats (Lamtai et al., 2020), which may be due to estrogen enhancing copper retention, making females more susceptible to its neurotoxic effects (Amtage et al., 2014). Therefore, further research is necessary to clarify the potential gender differences in the effects of copper homeostasis on neurocognitive function.

Lp(a) is a distinctive hepatic lipoprotein (Kamstrup, 2021), and there is evidence indicating a high correlation between serum Lp(a) levels and the severity of AD (Larsson et al., 2020; Merched et al., 2000), which suggests that clinical measurement of Lp(a) could be utilized for assessing the risk and severity of AD. Nevertheless, the relationship between Lp(a) and AD remains elusive. Some studies have demonstrated a significant positive correlation between serum concentrations of Lp(a) and increased risk of AD (Solfrizzi et al., 2002), while some studies have indicated a negative correlation (Gong et al., 2022), and others have found no significant difference in Lp(a) concentrations between AD patients and healthy controls (Ray et al., 2013; Ban et al., 2009). This current study reveals that the Lp(a) concentration in the AD patient group is higher than that in the control group, implying a potential positive correlation between Lp(a) and the risk of AD onset. However, due to the small sample size of this study, the statistical results may be subject to certain biases. Therefore, whether reducing Lp(a) levels would increase the risk of dementia requires further investigation with an expanded sample size (Larsson et al., 2020).

Immunocytes, particularly microglia and astrocytes, play a pivotal role in the pathogenesis of AD by modulating neuroinflammation (Princiotta Cariddi et al., 2022), Aβ clearance (Ennerfelt et al., 2022; Hur et al., 2020), and tau pathology (Rajesh and Kanneganti, 2022). Impaired immunocyte function can lead to reduced phagocytosis and accumulation of Aβ, while their activation results in the release of pro-inflammatory cytokines, neuronal damage, and disruption of synaptic integrity. Additionally, this activation may facilitate the spread of pathological Tau, induce neuronal apoptosis, and accelerate the decline in cognitive abilities in AD patients (da Mesquita et al., 2018). Recent investigative findings suggest that γδ-T cells, functioning as potential regulatory or pathogenic entities, are capable of infiltrating the brain (Aliseychik et al., 2020). During the early stages of AD, the IL-17 produced by γδ-T cells accumulates in the central nervous system in significant quantities and persists throughout the disease’s progression. IL-17 can cause in synaptic dysfunction and impairments in short-term memory in AD mouse models, which neutralization was sufficient to rescue Aβ-induced neuroinflammation and hippocampal glutamatergic dysfunction in early stages of disease with a mechanism that is independent of Aβ and Tau pathology or blood–brain barrier (BBB) disruption (Brigas et al., 2021; Cristiano et al., 2019). This is consistent with the findings of the current study, which observed a high enrichment of γδ-T cells in AD, potentially offering new avenues for the diagnosis and therapeutic intervention of AD.

In the analysis of the correlation between *FDX1* and immune cells in AD, we found that *FDX1* is closely associated with the infiltration of four types of immune cells, among which M0 macrophages were the most strongly associated. According to research reports, Chronic M0-differentiation may lead Triggering Receptor Expressed on Myeloid Cells 2 (TREM2), which serves as a putative therapeutic target for AD, to increased synthesis in AD-derived cells (Cosma et al., 2023). This suggests that the high expression of *FDX1* is correlated with immune pathways (Wang et al., 2024), and may participate in the occurrence and development of AD by altering the composition of the immune microenvironment.

There have been reports on the development of functional drugs targeting cuproptosis pathways and their application in cancer (Tong et al., 2022), but the role of cuproptosis and its related genes in AD has not been thoroughly studied. Therefore, developing new therapies for AD based on the cuproptosis is of great significance and deserves further investigation.

However, this study has certain limitations. Firstly, our study used a public dataset with limitations, which sample size is mainly composed of gene expression data, omitting other biological data like proteomics or metabolomics. Sample diversity and platform differences could bias our analysis and affect the findings’ generalizability. Diverse samples and multi-omics data should be utilized in future research to better understand disease mechanisms. Secondly, the sample size for clinical validation is relatively small, which may limit the universality and reliability of the research outcomes. Further validation and exploration are needed through the expansion of sample size and *in vitro* experiments. Thirdly, whether *FDX1* exclusively affects AD through the cuproptosis and the specific effects on AD after cuproptosis in neurons remain to be explored further.

## Conclusion

The key gene *FDX1* involved in cuproptosis is highly expressed in neurons and peripheral blood of AD, and it can affect the status of neurons by regulating the expression of *DLAT* and *DLST*. Thus, we speculate that *FDX1* may participate in the occurrence and development of AD through the cuproptosis. Exploring how to reduce *FDX1* expression to inhibit cuproptosis in neurons may become a new strategy for developing anti-AD drugs or delaying the progression of AD.

## Data Availability

The original contributions presented in the study are included in the article/supplementary material, further inquiries can be directed to the corresponding authors.

## Footnotes

^1^ https://www.ncbi.nlm.nih.gov/geo/

^2^ http://genemania.org/
